# Novel sex‐specific candidate loci associated with Alzheimer disease identified through sex‐aware multi‐ancestry genome‐wide meta‐analysis

**DOI:** 10.1002/alz70855_106407

**Published:** 2025-12-24

**Authors:** Brian W Kunkle, Lissette Gomez, Giuseppe Tosto, Omar Garcia Rodriguez, Susan H. Slifer, Kara L Hamilton‐Nelson, Adam C. Naj, Nicholas A. Kushch, Lily Wang, Juan I Young, Sven‐Thorsten Dietrich, Amanda Kuzma, Laura B Cantwell, Otto Valladares, William S Bush, Timothy J. Hohman, Logan Dumitrescu, Ryan Dacey, Nicholas R. Ray, Christiane Reitz, Li‐San Wang, Lindsay A. Farrer, Richard Mayeux, Jonathan L Haines, Margaret Pericak‐Vance, Gerald D. Schellenberg, Eden R. Martin, Alzheimer's Disease Genetics Consortium (ADGC), Alzheimer's Disease Sequencing Project (ADSP)

**Affiliations:** ^1^ John P. Hussman Institute for Human Genomics, Miller School of Medicine, University of Miami, Miami, FL, USA; ^2^ Dr. John T. Macdonald Foundation Department of Human Genetics, University of Miami Miller School of Medicine, Miami, FL, USA; ^3^ John P. Hussman Institute for Human Genomics, University of Miami Miller School of Medicine, Miami, FL, USA; ^4^ Department of Neurology, College of Physicians and Surgeons, Columbia University, and the New York Presbyterian Hospital, New York, NY, USA; ^5^ University of Miami Miller School of Medicine, Miami, FL, USA; ^6^ Penn Neurodegeneration Genomics Center and Department of Biostatistics, Epidemiology, and Informatics, University of Pennsylvania, Philadelphia, PA, USA; ^7^ Department of Public Health Sciences, University of Miami Miller School of Medicine, Miami, FL, USA; ^8^ Penn Neurodegeneration Genomics Center, Department of Pathology and Laboratory Medicine, Perelman School of Medicine, University of Pennsylvania, Philadelphia, PA, USA; ^9^ University of Pennsylvania Perelman School of Medicine, Philadelphia, PA, USA; ^10^ Department of Population and Quantitative Health Sciences, Institute for Computational Biology, Case Western Reserve University, Cleveland, OH, USA; ^11^ Vanderbilt Genetics Institute, Vanderbilt University Medical Center, Nashville, TN, USA; ^12^ Department of Medicine (Biomedical Genetics), Boston University Chobanian & Avedisian School of Medicine, Boston, MA, USA; ^13^ Taub Institute for Research on Alzheimer's Disease and the Aging Brain, Columbia University, New York, NY, USA; ^14^ Gertrude H. Sergievsky Center, Taub Institute for Research on the Aging Brain, Departments of Neurology, Psychiatry, and Epidemiology, College of Physicians and Surgeons, Columbia University, New York, NY, USA; ^15^ Penn Neurodegeneration Genomics Center, Department of Pathology and Laboratory Medicine, University of Pennsylvania Perelman School of Medicine, Philadelphia, PA, USA; ^16^ Biomedical Genetics, Department of Medicine, Boston University Medical School, Boston, MA, USA; ^17^ Taub Institute for Research on Alzheimer's Disease and the Aging Brain, Columbia University, New York, NY, USA; ^18^ John P. Hussman Institute for Human Genomics, University of Miami, Miami, FL, USA; ^19^ Penn Neurodegeneration Genomics Center, Perelman School of Medicine, University of Pennsylvania, Philadelphia, PA, USA

## Abstract

**Background:**

Sex differences in progression and pathology of Alzheimer's disease (AD) suggest sex‐specific factors influencing its development. While studies have established *APOE*‐genotype as contributing to AD risk differently in men and women, few have searched for additional genetic sex differences in AD. To identify sex‐specific AD genetic associations, we conducted genome‐wide sex‐aware meta‐analyses in the Alzheimer's Disease Genetics Consortium (ADGC) and Alzheimer's Disease Sequencing Project (ADSP) datasets.

**Method:**

Sex‐interaction and sex‐stratified analyses were performed in multi‐ancestry genome‐wide imputed AD datasets from the ADGC (*N* = 25,284 cases, 60% female and 33,455 controls, 63% female; ancestry/ethnicity distribution: 63.7% European, 15.6% African, 15.2% Hispanic/Latino, 5.5% Asian). STAAR aggregation‐based rare‐variant testing was also conducted in coding and non‐coding genomic regions on an ancestrally diverse sample of 8,697 AD cases and 14,758 controls with whole‐genome sequencing from the ADSP.

**Result:**

Cross‐ancestry sex‐stratified analyses identified several loci with evidence of interaction (*p* <0.05) and suggestive significance in one sex (*p* <5x10‐6) and not the other (*p* >0.05). These include male specific variants in *STXBP6, MAP4K5* and the known AD locus *PICALM*. Genetic loci associated in females and not males include *NPAS3, ZNF438* and a genome‐wide result in *NECTIN2* near the *APOE* locus. Top ancestry‐specific results include associations at *COL4A2* in Asian‐ancestry males and *C16orf96* in African‐ancestry females.

Cross‐ancestry rare‐variant aggregation‐based testing revealed four novel genome‐wide significant associations in females including with missense variants in *SH3BP1*. Five population specific associations were also discovered including with missense variants in *SERTAD4* in a genetically‐defined Hispanic/Amerindian/African ancestral population cluster and promoter variants of *PSMA5* in a European population cluster. Seven male‐specific effects were also discovered including genome‐wide significant cross‐ancestry associations with an enhancer region of *MORC1* and a promoter of *ITPKA*.

**Conclusion:**

We identified sex‐specific AD associations at loci with AD‐relevant genes including *STXBP6* (involved in AD relevant processes such as endolysosomal transport and synaptic transmission), *NPAS3* (neurogenesis), *COL4A2* (cerebral vasculature), *ITPKA* (learning/memory processes), *PAQR3* (cholesterol homeostatis and neuronal function), and *MORC1* (recently associated in a UK Biobank exome‐wide sequencing study of dementia (Zhang et al. Alz & Dementia 2024). Understanding the nature of these associations could help explain sex differences in risk and progression for AD.

